# Cytokines and growth factors cross-link heparan sulfate

**DOI:** 10.1098/rsob.150046

**Published:** 2015-08-12

**Authors:** Elisa Migliorini, Dhruv Thakar, Jens Kühnle, Rabia Sadir, Douglas P. Dyer, Yong Li, Changye Sun, Brian F. Volkman, Tracy M. Handel, Liliane Coche-Guerente, David G. Fernig, Hugues Lortat-Jacob, Ralf P. Richter

**Affiliations:** 1 Université Grenoble Alpes, Departement de Chimie Moléculaire (DCM), Grenoble, France; 2 CNRS, DCM, Grenoble, France; 3 CIC biomaGUNE, San Sebastian, Spain; 4 Department of Biophysical Chemistry, University of Heidelberg, Heidelberg, Germany; 5 Université Grenoble Alpes, Institut de Biologie Structurale (IBS), Grenoble, France; 6 CNRS, IBS, Grenoble, France; 7 CEA, IBS, Grenoble, France; 8 University of California, San Diego, Skaggs School of Pharmacy and Pharmaceutical Sciences, La Jolla, CA, USA; 9 Department of Biochemistry, Institute of Integrative Biology, University of Liverpool, Liverpool, UK; 10 Department of Biochemistry, Medical College of Wisconsin, Milwaukee, WI, USA; 11 Max Planck Institute for Intelligent Systems, Stuttgart, Germany

**Keywords:** heparan sulfate, glycosaminoglcyan, extracellular matrix, chemokine, growth factor, cytokine

## Abstract

The glycosaminoglycan heparan sulfate (HS), present at the surface of most cells and ubiquitous in extracellular matrix, binds many soluble extracellular signalling molecules such as chemokines and growth factors, and regulates their transport and effector functions. It is, however, unknown whether upon binding HS these proteins can affect the long-range structure of HS. To test this idea, we interrogated a supramolecular model system, in which HS chains grafted to streptavidin-functionalized oligoethylene glycol monolayers or supported lipid bilayers mimic the HS-rich pericellular or extracellular matrix, with the biophysical techniques quartz crystal microbalance (QCM-D) and fluorescence recovery after photobleaching (FRAP). We were able to control and characterize the supramolecular presentation of HS chains—their local density, orientation, conformation and lateral mobility—and their interaction with proteins. The chemokine CXCL12*α* (or SDF-1*α*) rigidified the HS film, and this effect was due to protein-mediated cross-linking of HS chains. Complementary measurements with CXCL12*α* mutants and the CXCL12*γ* isoform provided insight into the molecular mechanism underlying cross-linking. Fibroblast growth factor 2 (FGF-2), which has three HS binding sites, was also found to cross-link HS, but FGF-9, which has just one binding site, did not. Based on these data, we propose that the ability to cross-link HS is a generic feature of many cytokines and growth factors, which depends on the architecture of their HS binding sites. The ability to change matrix organization and physico-chemical properties (e.g. permeability and rigidification) implies that the functions of cytokines and growth factors may not simply be confined to the activation of cognate cellular receptors.

## 1. Background

Heparan sulfate (HS) is a linear polysaccharide made of variably sulfated repeating disaccharide units. Attached to extracellular matrix or cell-surface proteins (HS proteoglycans, HSPGs), it pervades the intercellular space of many tissues and the periphery of virtually all mammalian cells. HS binds many soluble extracellular signalling molecules such as growth factors and chemokines, and these interactions are known to be important for various physiological and pathological processes [1–4] including organogenesis and growth control [5,6], cell adhesion [7] and signalling [8], inflammation [9], tumour development [10] and interactions with pathogens [11].

Past studies have revealed how HS–protein interactions determine protein function. For example, HS (as well as the highly sulfated analog heparin) plays a role in the specificity and control of the engagement of fibroblast growth factors (FGFs) with their cell-surface receptors, through the formation of stable ternary complexes [12], thus modulating cell signalling. The binding of chemokines to HS in the extracellular space, on the other hand, enables the formation of chemokine gradients [13], thus providing directional cues and guiding the migration of appropriate cells in the context of their inflammatory, developmental and homeostatic functions.

By contrast, very little is known about the effect of signalling proteins on HS and HSPGs. HS chains are typically a few tens of nanometres in length [14] and, thus, possess multiple binding sites enabling simultaneous binding of several proteins [15]. These interactions will influence the molecular structure of individual HS chains. Moreover, they may also profoundly affect the supramolecular organization of HS in the extracellular space. Such long-range effects have hitherto been difficult to test, because of the lack of appropriate structural and biochemical methods.

Here, we demonstrate that several soluble extracellular signalling proteins can effectively cross-link HS. To this end, we developed an *in vitro* binding assay that is based on films of surface-grafted HS chains, as a well-defined model of HS-rich pericellular or extracellular matrix [7], and a combination of two biophysical analysis techniques: quartz crystal microbalance (QCM-D) and fluorescence recovery after photobleaching (FRAP). These techniques provide insight into the binding of proteins to the HS film, and the concomitant changes in film morphology and HS chain mobility. Through the analysis of a set of proteins and their mutants—including chemokines, cytokines and growth factors—with this assay, we identify molecular features that determine the HS cross-linking propensity of extracellular signalling proteins. The ability to cross-link, and thus to change matrix organization and physico-chemical properties, implies that the functions of these proteins may not simply be confined to the activation of cognate cellular receptors, and we discuss possible physiological implications.

## 2. Material and methods

### 2.1. Buffer

The working buffer used for all measurements contained 10 mM HEPES (Fisher, Illkirch, France) at pH 7.4 and 150 mM NaCl (Sigma-Aldrich, Saint-Quentin Fallavier, France).

### 2.2. Heparan sulfate and proteins

The HS polysaccharide derived from porcine intestinal mucosa (Celsus Laboratories, Cincinnati, OH, USA) was found to have an average molecular weight of 12 kDa and a polydispersity of 1.6 [16]. Size-uniform HS oligosaccharides from hexasaccharide (degree of polymerization, dp6) to dodecasaccharide (dp12) were derived from this source, as previously described [17]. HS was conjugated with biotin through an oligoethylene glycol (OEG) linker of approximately 1 nm length, site-specifically attached to the reducing end by oxime ligation. In contrast to the conventionally used hydrazone ligation, oxime ligation produces conjugates that are stable for many weeks in aqueous solution [18]. HS conjugates were stored at a concentration of 10 mg ml^−1^ at −20°C until further use.

Recombinant CXCL12*α* (amino acids 1 to 68; 8.1 kDa) was prepared as previously described [19]. A truncated CXCL12*α* construct (amino acids 5 to 67; 7.4 kDa [20]) was produced by solid-phase peptide synthesis, as previously reported [4,15]. An I55C/L58C mutant of CXCL12*α* with reduced dimerization propensity (‘partial monomer’) was prepared as previously described [21]. An L36C/A65C mutant of CXCL12*α* in which the introduced cysteines promote the formation of dimers (‘locked dimer’) was prepared, as described in Veldkamp *et al.* [22]. The cDNA of murine CXCL12*γ* was inserted in a pET-17b vector (Novagen, Merck Chemical Ltd., Nottingham, UK) between NdeI and SpeI restriction sites, checked by DNA sequencing, and the protein (11.6 kDa) was produced by recombinant expression in *Escherichia coli* strain BL21 Star DE3, as previously reported [23]. Interferon (IFN)*γ* (17 kDa) was produced by recombinant expression in *E. coli* strain BL21 Star DE3 using a pET-11a vector (Novagen), as previously reported [24]. Recombinant FGF-2 (18 kDa) and FGF-9 (26 kDa) were obtained by expression in C41 *E. coli* cells using pET-14b and pET-M11 for vectors (Novagen), respectively, as described by Xu *et al.* [25].

Lyophilized streptavidin (SAv), fluorescently labelled SAv (fl-SAv; with atto565) and bovine serum albumin (BSA) were obtained from Sigma-Aldrich. All proteins were stored in working buffer at −20°C until further use. Thawed protein solutions were used within 5 days.

### 2.3. Surfaces and surface funtionalization with a biotin-displaying and otherwise inert background

QCM-D sensors with gold (QSX301) and silica (QSX303) coatings (Biolin Scientific, Västra Frölunda, Sweden) were used as is. Glass coverslips (24 × 24 mm^2^; Menzel-Gläser, Braunschweig, Germany) for FRAP assays were cleaned by immersion in freshly prepared piranha solution (i.e. a 1 : 3 (*v*/*v*) mixture of H_2_O_2_ (Fisher Scientific) and concentrated H_2_SO_4_ (Sigma-Aldrich)) for 1 h, rinsing with ultrapure water and blow-drying with N_2_. All substrates were exposed to UV/ozone (Jelight Company, CA, USA) for 10 min prior to use.

Gold surfaces were functionalized with biotin-displaying monolayers of OEG as previously described [7]. Briefly, the gold-coated surfaces were immersed overnight in an ethanolic solution of OEG disulfide and biotinylated OEG thiol (Polypure, Oslo, Norway), at a total concentration of 1 mM and a molar ratio of thiol equivalents of 999 : 1.

Silica (for QCM-D) and glass (for FRAP) surfaces were functionalized with biotin-displaying supported lipid bilayers (SLBs) by the method of vesicle spreading, as described in detail elsewhere [26]. Briefly, the surfaces were exposed for 30 min to small unilamellar vesicles, made from a mixture of dioleoylphosphatidylcholine (DOPC) and dioleoylphosphatidylethanolamine-CAP-biotin (DOPE-CAP-b) (Avanti Polar Lipids, Alabaster, AL, USA) at the desired molar ratio (99.5 : 0.5 or 95 : 5) at a total concentration of 50 µg ml^−1^ in working buffer supplemented with 2 mM CaCl_2_ (VWR International, Leuven, Belgium).

### 2.4. Assembly of HS films

Biotin-displaying surfaces were further functionalized for studies of protein interactions with well-defined HS films, as described in detail earlier [7]. Briefly, the surfaces were first exposed to SAv, to form a SAv-monolayer, and then to biotinylated HS (b-HS), to form a molecular film of HS that is site-specifically attached through the reducing end to the surface. This mode of attachment avoids any perturbation of protein–HS interactions through chemical modifications along the HS chain [27,28]. Sample concentrations and incubation times were chosen such that binding either saturates or equilibrates, unless otherwise stated.

### 2.5. Quartz crystal microbalance with dissipation monitoring

Quartz crystal microbalance with dissipation monitoring (QCM-D) measurements were performed, as previously described [7]. QCM-D measures changes in frequency, Δ*f*, and in dissipation, Δ*D*, of a quartz sensor upon interaction of molecules with its surface. Measurements were performed with a Q-Sense E4 system equipped with Flow Modules (Biolin Scientific) with a flow rate of typically 10 µl min^−1^ and at a working temperature of 24°C. QCM-D data were collected at six overtones (*n* = 3, 5, 7, 9, 11, 13, corresponding to resonance frequencies of approximately 15, 25, 35, 45, 55, 65 MHz). For the sake of simplicity, only changes in dissipation and normalized frequency, Δ*f* = Δ*f_n_*/*n*, of the third overtone (*n* = 3) are presented. Any other overtone would have provided comparable information.

A viscoelastic model [29], implemented in the software QTM (Diethelm Johannsmann, Clausthal University of Technology; http://www2.pc.tu-clausthal.de/dj/software_en.shtml), was used to quantify the thickness *d* and viscoelastic properties of HS films from QCM-D data. Details of the fitting procedure are described elsewhere [30]. We parametrized viscoelastic properties in terms of the elastic and viscous compliances *J*′ and *J*″ at a reference frequency of *f* = 15 MHz (i.e. close to the resonance frequency at *n* = 3). *J*′ and *J*″ are measures for the softness of the film. The elastic compliance can also be estimated directly from the QCM-D responses for the film through the approximate relationship Δ*D*/(−Δ*f*) = 4*πnη*_l_*ρ*_l_/*ρ* × *J*′, where *η*_l_ = 0.89 mPa·s and *ρ*_l_ = 1.0 g cm^−3^ are the viscosity and density of the aqueous bulk solution, respectively, and *ρ* ≈ 1.0 g cm^−3^ is the film density [31].

### 2.6. Fluorescence recovery after photobleaching

For fluorescence recovery after photobleaching (FRAP) assays, cleaned glass coverslips were attached, using a bi-component glue (Picodent, Wipperfürth, Germany), to a custom-built teflon holder, thus forming the bottom of four identical wells with a volume of 50 µl each. All surface functionalization steps were performed in still solution. To remove excess sample after each incubation step, the content was diluted by repeated addition of a twofold excess of working buffer and removal of excess liquid until the concentration of the solubilized sample, estimated from the extent of dilution, was below 10 ng ml^−1^. Repeated aspiration and release ensured homogenization of the liquid volume at each dilution step. Care was taken to keep the substrates wet at all times.

FRAP measurements were performed with a confocal laser scanning microscope (LSM 700, Zeiss, Germany) using a laser with 555 nm wavelength, a plan-apochromat 63×/1.4 oil immersion objective and a fully opened pinhole (1 mm diameter). fl-SAv, attached to biotin-displaying SLBs, was used as a fluorophore to report on the lateral mobility of SAv-bound HS.

After acquiring three pre-bleach images, a circular region with a radius of 10 µm in the centre of the imaged area was bleached through exposure for approximately 20 s to high laser intensity; approximately 80% bleaching in the centre of the exposed area was achieved. The fluorescence recovery due to lateral diffusion of bleached and unbleached fl-SAv was monitored through acquisition of post-bleach images over a period of typically 10 min.

The images acquired using this protocol were then analysed by ‘time-resolved profile analysis’, a custom-made algorithm [32] implemented in Matlab (MathWorks, MA, USA). Briefly, each post-bleach fluorescence image was first corrected for background fluorescence, spatial aberrations and intensity fluctuations and then radially averaged. The radial intensity profiles thus obtained were compared with numerical solutions of a diffusion equation, where the first post-bleach image defined the initial conditions for the diffusion process. A lateral diffusion model with one mobile fraction and one immobile fraction was found to reproduce our data well. This model has two independent fitting parameters, namely the size and diffusion constant of the mobile fraction. These were computed through global minimization of the root-mean-square differences between numerical predictions and all post-bleach profiles.

## 3. Results

**Figure 1.**
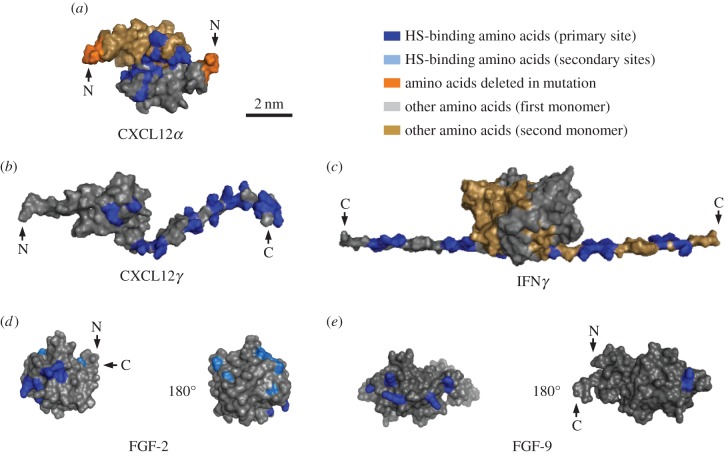
Structures of soluble extracellular signalling proteins used in this study. Structures are surface plots, all drawn at the same scale (scale bar indicated in (*a*)). Amino acids known to contribute to primary and secondary HS binding sites are shown in dark and light blue, respectively; the remaining protein surfaces are coloured in grey, or in light brown for the second monomer in the structures of homodimers; the position of selected N or C terminals are marked with an arrow. CXCL12*α* (*a*) is shown as a homodimer associated through β-sheets (PDB code: 1QG7, where missing residues were added as described in [17]) with its reported HS-binding amino acids [15,17,33] and the first four amino acids, lacking in the CXCL12*α*(5–67) mutant, indicated (orange). CXCL12*γ* (*b*) was constructed from a CXCL12*α* monomer and the additional 30 amino acid long N-termini modelled as previously reported [23]. IFN*γ* (*c*; PDB code: 1HIG [34]) is shown as a homodimer with the C-termini (residues 120–143, absent in the structure) built as extended β-strands. FGF-2 (*d*; PDB code: 1FQ9 [35]) and FGF-9 (*e*; PDB code: 1IHK [36]) are shown as monomers with their known HS binding sites, i.e. three sites for FGF-2 [37] and a single, extended site for FGF-9 [25].

**Figure 2.**
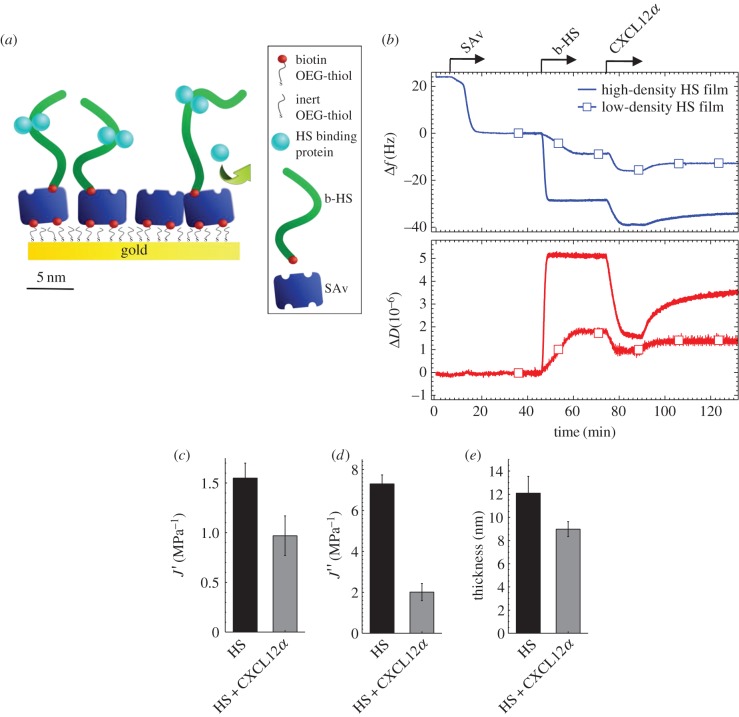
Design of HS films and effect of CXCL12*α* binding. (*a*) Schematic representation with the relative sizes of all molecules approximately drawn to scale. HS is biotinylated at the reducing end for oriented and specific immobilization on streptavidin (SAv). SAv is specifically bound to a gold-supported monolayer of thiolated oligo(ethylene glycol) (OEG) exposing terminal biotin. (*b*) Surface functionalization and CXCL12*α* binding followed by QCM-D (frequency shifts, Δ*f*, dissipation shifts, Δ*D*). Start and duration of incubation steps with different samples are indicated by an arrow; during all other times, the surface was exposed to buffer. SAv was first incubated at 1 µg ml^−1^ and then at 20 µg ml^−1^ and responses are consistent with the formation of a dense protein monolayer [7]. b-HS was incubated either at 50 µg ml^−1^ to saturation (‘high-density HS films’, curves without symbols) or at 1 µg ml^−1^ for 15 min to reach about 30% of maximal coverage (‘low-density HS films’, curves with square symbols). CXCL12*α*, incubated at 0.64 µM, induced dissipation decreases for both HS densities, indicating rigidification of the hydrated HS film upon chemokine binding. (*c*) Elastic compliance *J*′, viscous compliance *J*″ and thickness of HS films obtained from QCM-D data for high-density HS films, bare and with CXCL12*α* at binding equilibrium. Data correspond to mean and standard error of the mean from three independent experiments. All parameters decreased upon CXCL12*α* incubation, confirming film rigidification and compaction.

**Figure 3.**
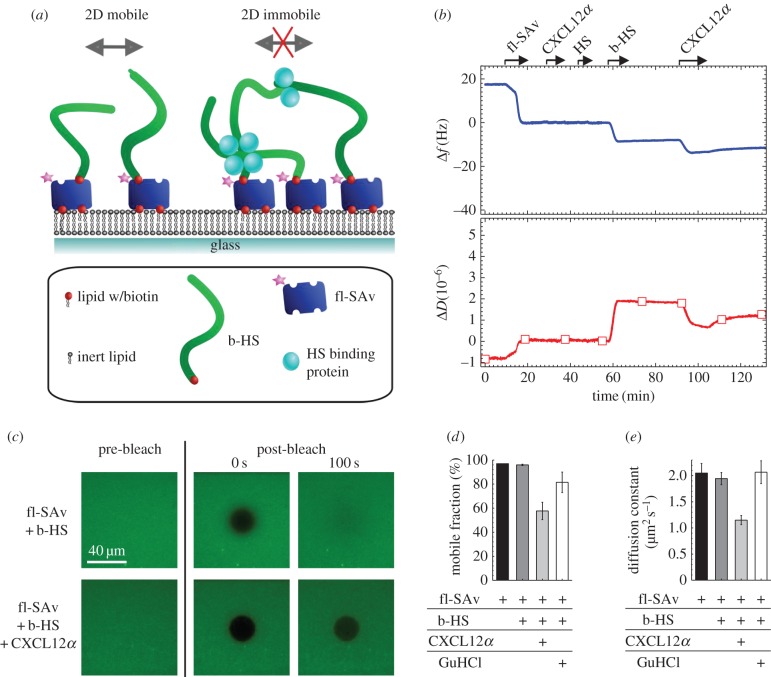
FRAP confirms CXCL12*α*-mediated cross-linking of HS films. (*a*) Schematic representation of HS films used for FRAP experiments. b-HS, anchored to fl-SAv, can diffuse along the surface (2D mobile) thanks to a fluid biotin-presenting supported lipid bilayer (left). Cross-linking, mediated by HS-binding proteins, is expected to lead to a reduction of HS (and hence fl-SAv) lateral mobility (right). (*b*) Surface functionalization and CXCL12*α* binding followed by QCM-D. Start and duration of each incubation step with different samples are indicated by an arrow; during all other times, the surface was exposed to buffer. fl-SAv was incubated at 20 µg ml^−1^ until saturation; the low percentage of biotinylated lipids (0.5%) limits fl-SA binding to a sub-monolayer. CXCL12*α* (0.64 µM) and 50 µg ml^−1^ biotin-free HS produced no measurable response, confirming that the fluorescent label does not induce any non-specific binding. The QCM-D responses for b-HS (incubated at 50 µg ml^−1^ to saturation) and for CXCL12*α* (incubated at 0.64 µM) were comparable with the low-density HS films shown in figure 2*b*. (*c*) Representative fluorescence micrographs demonstrating the FRAP assay to assess chemokine-mediated cross-linking. Recovery of the bleached spot is seen after 100 s for a bare HS film, but not for a CXCL12*α*-loaded HS film. (*d,e*) Quantitative analysis of FRAP data in terms of the mobile fraction (*d*) and its diffusion constant (*e*). Lateral mobility of fl-SAv was assessed in the absence of b-HS, after incubation with b-HS at saturation, after 15 min incubation of the HS film with chemokines (in the presence of 0.64 µM chemokines in solution) and after regeneration of the HS film by 2 M GuHCl. Data correspond to mean and standard error of the mean for three independent experiments.

We tested the effect of several extracellular signalling molecules on HS model matrices, namely the *α* and *γ* isoforms of the chemokine CXCL12, the cytokine IFN*γ* and the growth factors FGF-2 and FGF-9. These were selected based on their known affinity for HS and distinct structural features (figure 1). All proteins bind HS more strongly, or at least as strongly, as other glycosaminoglycans (GAGs) such as chondroitin sulfate or dermatan sulfate [23,25,38–41], suggesting that HS serves as their natural ligand in HS-rich extracellular matrices. CXCL12*α* forms homodimers through the association of β-sheets upon binding to HS, with the known HS binding site being located at the interface between the two monomers (figure 1*a*). CXCL12*γ* is distinct from CXCL12*α* in that it features flexible C-terminal extensions that are also involved in HS binding, and that it is not known to form β-sheet dimers (figure 1*b*). IFN*γ* is constitutively present as a homodimer which features a very extended HS binding surface on the flexible C-termini of the monomers (figure 1*c*). The FGFs are more compact. FGF-2 has three distinct HS binding sites (figure 1*d*) that are separated from each other by borders of negatively charged and hydrophobic residues. FGF-9, by contrast, features only one HS binding site (figure 1*e*). As HS matrix model, we employed films of HS chains grafted with the reducing end to a protein-repellant surface (figure 2*a*). QCM-D allows monitoring of HS film assembly and protein binding as well as analysis of film thickness and mechanical properties (figure 2). FRAP allows for the lateral mobility of HS chains to be probed (figure 3).

### 3.1. Design of HS model matrix

Our HS films present HS in an oriented manner and at controlled density (figure 2*a*) [7]. Gold supports were first coated with a monolayer of OEG exposing terminal biotin groups at controlled surface density. A monolayer of SAv was then formed and used to anchor HS through a biotin moiety that was conjugated to the GAG's reducing end [7]. Binding in this orientation effectively reproduces the attachment of HS to core proteins in HSPGs [42], and minimizes effects of biotin conjugation and surface-confinement on protein binding. The SAv-on-OEG film inhibits non-specific protein binding to the surface, i.e. measured responses are exclusively because of specific interactions.

QCM-D was used to validate correct assembly of the model surface and to characterize the effect of protein binding on HS films. The QCM-D response is sensitive to the amount of adsorbed ligand (including coupled solvent), with a negative frequency shift Δ*f* typically correlating with a mass increase, and to mechanical properties, as well as morphological features of the biomolecular film, typically reflected in the dissipation shift Δ*D* [31].

QCM-D responses upon sequential incubation of OEG monolayers with SAv and HS at saturation (figure 2*b*, curves without symbols; at 6–21 min and 46–61 min, respectively, as indicated by arrows on top of the graph) were consistent with the formation of a relatively rigid SAv-monolayer (i.e. with Δ*f* = −23 ± 1 Hz and a low dissipation shift, Δ*D* ≤ 0.3 × 10^−6^, at saturation) and a soft, hydrated HS layer (i.e. with Δ*f* = −28.5 ± 1.0 Hz and a high dissipation shift, Δ*D* = 5.0 ± 0.2 × 10^−6^, at saturation), respectively. As reported in our previous study [7], the frequency shift for such an HS film (henceforward called high-density HS film) corresponds to an areal mass density of 35.5 ± 2.2 ng cm^−2^, and to a water content of 96.9 ± 0.5%. In this earlier work, we had also estimated the mean distance between adjacent HS anchor sites to be 5 nm, consistent with the dimensions of SAv, and the mean length of the surface-bound HS chains to be 20 monosaccharides (or 10 nm); in this regard, we note that the mean length of surface-bound b-HS chains is shorter than the mean length in the solution from which they were bound, because shorter chains bind preferentially [7]. In essence these numbers indicate that, while there is plenty of space for small proteins to bind into the HS films, the pendant HS chains are long enough to make contact with their neighbours and cover the whole surface area.

### 3.2. Effect of CXCL12*α* binding on HS films

Exposure of the chemokine CXCL12*α* at a concentration of 0.64 µM to the high-density HS film generated a negative frequency shift (−9 ± 1 Hz; figure 2*b*, blue curve without symbols, 74 to 90 min), confirming CXCL12*α* binding. The concomitant change in dissipation was pronounced and negative (−3.8 ± 0.2 × 10^−6^; figure 2*b*, red curve without symbols). Such a QCM-D response provides a strong indication that the chemokine rigidifies the HS film. Quantitative analysis of the QCM-D data through viscoelastic modelling revealed decreases in the elastic compliance *J*′ and the viscous compliance *J*″ upon CXCL12*α* binding (figure 2*c*). *J*′ and *J*″ are physical parameters (elastic and viscous contributions, respectively) related to film softness, and their decrease thus confirms film rigidification. This analysis also revealed that the protein induces a moderate decrease in film thickness (figure 2*c*). Upon subsequent rinsing in buffer, frequency and dissipation increased slowly, but did not return to the level of the virgin HS film (figure 2*b*, curves without symbols; from 89 min), demonstrating that some, but not all CXCL12*α* is released over experimentally accessible time scales, and that the HS film partially recovers its original morphology.

To test if the protein-induced morphological changes depend on HS surface density, we repeated the QCM-D assay at reduced HS surface coverage (figure 2*b*, curves with square symbols). To this end, b-HS was incubated at a lower solution concentration (1 µg ml^−1^) and binding was interrupted after 15 min (figure 2*b*, 46 to 61 min). The frequency shift for HS (−8 ± 1 Hz) in this case (henceforward called low-density HS film) corresponds to an areal mass density of 12.0 ± 0.5 ng cm^−2^ and an average distance between adjacent HS anchors of about 10 nm, according to previously reported estimates [7]. It is thus likely that most HS chains can make contacts with their neighbours even for low-density HS chains. CXCL12*α* induced a clear (albeit smaller) decrease in dissipation (figure 2*b*, 74 to 90 min), i.e. film rigidification also occurred on low-density HS films.

### 3.3. Effect of CXCL12*α* binding on HS chain mobility

We hypothesized that the rigidification and thinning of HS films is due to cross-linking of HS chains by the chemokine. However, an alternative explanation could be that individual HS chains wrap around CXCL12*α* molecules, thereby stiffening the film and reducing the film thickness without generating any inter-chain cross-links. To distinguish between these two scenarios, we tested how the chemokine affects the lateral mobility of HS chains.

To this end, we used a modified model surface in which the gold-supported OEG monolayer was replaced by a silica- or glass-supported lipid bilayer (SLB; figure 3*a*). The oriented immobilization of HS at controlled densities is retained on these surfaces and the SAv-on-SLB film is also effectively passivating against non-specific binding of proteins [7]. SLBs are distinct, however, in that they provide a fluid surface on which SAv, and the SAv-bound b-HS, have the freedom to move laterally (schematically shown in figure 3*a*).

The lateral mobility was probed by FRAP, using fluorescently labelled SAv (fl-SAv) as b-HS anchors. In this method, a limited surface area is rapidly bleached and diffusion of fluorescent molecules into (and bleached molecules out of) the bleached area is subsequently monitored.

We verified correct surface functionalization by QCM-D (figure 3*b*). The fraction of biotinylated lipids used to form SLBs was adjusted to 0.5% such that incubation of fl-SAv at saturation (figure 3*b*, 10 to 20 min) led to a partial protein monolayer, in which the SAv molecules diffused freely, i.e. without being appreciably hindered by two-dimensional crowding. The fluorescent label did not induce any non-specific binding of CXCL12*α* or HS (figure 3*b*, 29 to 37 min and 44 to 51 min, respectively). The shifts in frequency (−9 ± 1 Hz) and dissipation (2 ± 0.2 × 10^−6^) for incubation of b-HS at saturation (figure 3*b*, 58 to 68 min) were comparable with the low-density HS film shown in figure 2*b*. Moreover, the QCM-D responses upon subsequent binding of CXCL12*α* (figure 3*b*, 92 to 105 min) were also similar to those observed in figure 2*b*. This indicates that the FRAP measurements can be directly correlated with QCM-D measurements on low-density HS films.

The representative fluorescence micrographs in figure 3*c* demonstrate close-to-complete recovery of virgin b-HS films within 100 s, confirming that fl-SAv with HS is indeed laterally mobile, as desired. By contrast, the bleached spot remained clearly visible after 100 s when CXCL12*α* was added to the HS film. Radially averaged fluorescence intensity profiles were computed from time-lapse series of micrographs after photobleaching, and analysed to quantify lateral mobility. To this end, the pool of fl-SAv was assumed to be distributed in two distinct fractions, one immobile and the other laterally mobile with a given diffusion constant. The size of the mobile fraction and its diffusion constant are shown in figure 3*d,e*. These quantitative results confirm that virtually all (i.e. ≥ 95%) fl-SAv in a virgin SAv-monolayer was mobile, and that the mobility was unaffected by the presence of b-HS. In the presence of CXCL12*α*, 40% of the fl-SAv became effectively immobilized, and additionally, the diffusion constant of the retained mobile fraction was strongly reduced (by 45%). These data provide evidence that CXCL12*α* impedes lateral motion of HS and its fl-SAv anchor, and we propose that this immobilization is the consequence of CXCL12*α*-mediated HS cross-linking.

After treatment with 2 M GuHCl, which we know effectively releases all CXCL12*α* from HS while keeping the HS film intact [7], the mobile fraction and its diffusion constant largely returned to the values observed for a virgin HS film. This confirms that HS mobility is restored upon chemokine release, i.e. the cross-linking is reversible and requires the presence of the chemokine. The mobile fraction though remained marginally reduced, indicating that a small fraction of fl-SAv remains permanently immobile upon GuHCl treatment. Most probably, the lack of complete regeneration is due to a weak yet irreversible perturbation of the fl-SAv film by GuHCl: detailed inspection of the fluorescent micrographs after GuHCl treatment revealed bright spots that we believe are fl-SAv aggregates.

### 3.4. Effect of CXCL12*α* mutations on HS cross-linking

CXCL12*α* is known to form β-sheet dimers (figure 1*a*) upon binding to HS [15]. To test if this oligomerization is involved in HS cross-linking, we additionally tested two CXCL12*α* constructs with point mutations that leave the ternary structure of CXCL12*α* essentially intact, but alter the ability of the protein to form β-sheet dimers: L36C/A65C mutations result in inter-molecular disulfide bonds and formation of a ‘locked dimer’ [22] while I55C/L58C mutations promote an intra-molecular disulfide bond and formation of a ‘partial monomer’ with a reduced propensity to form dimers [21].

**Figure 4.**
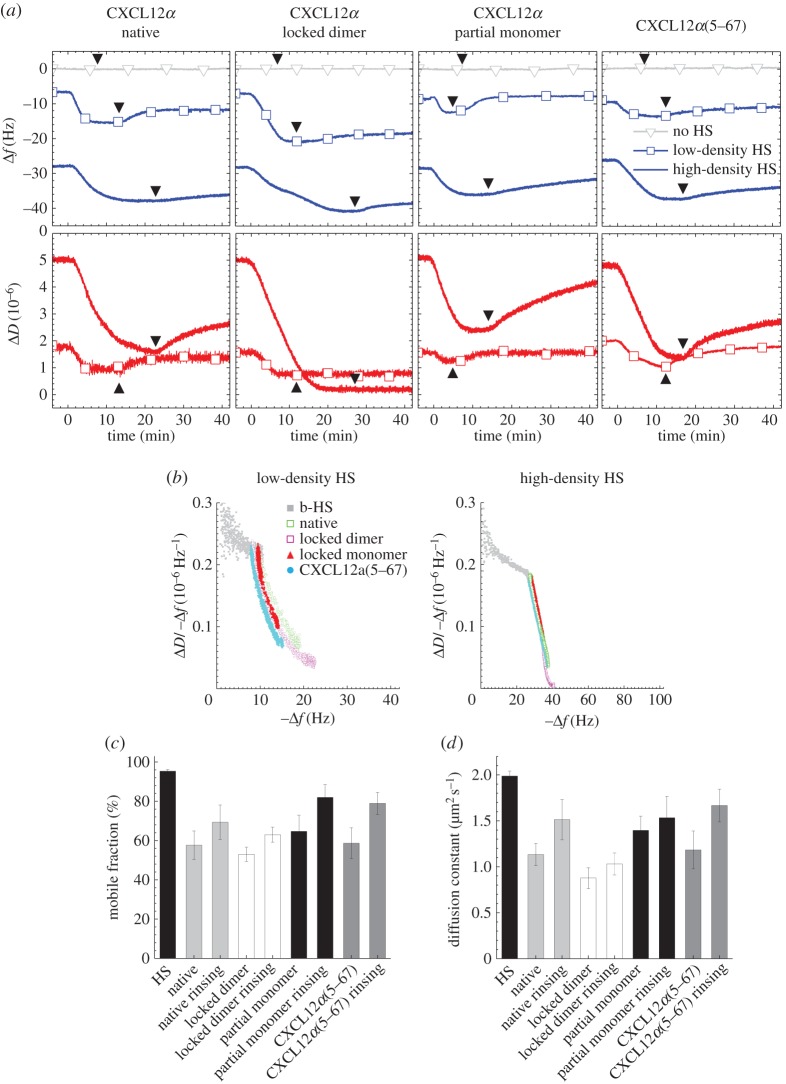
Dimerization and N-terminal lysine are dispensable for HS cross-linking. (*a*) QCM-D data for the binding of selected CXCL12*α* constructs to low-density (curves with square symbols) and high-density (curves without symbols) HS films on SAv on OEG monolayers. As in figure 2*b*, Δ*f* and Δ*D* are shown relative to SAv-coated surfaces before b-HS binding, yet b-HS binding is not explicitly shown. All samples were injected at 0 min and a concentration of 0.64 µM monomer equivalents; arrowheads indicate the start of rinsing in working buffer. Protein binding was also tested on SAv-monolayers without HS (grey curves with triangle symbols; only shown for Δ*f*) to confirm absence of non-specific binding. Frequency shifts at equilibrium and unbinding curves after rinsing differed between CXCL12*α* constructs, indicating that their binding affinities are distinct. However, all constructs induced dissipation decreases on low- and high-density films, indicating HS film rigidification. (*b*) Parametric plot of Δ*D*/−Δ*f* for the protein-loaded HS film (a relative measure for film softness) versus –Δ*f* for protein binding (a relative measure for protein surface density) for the binding data on low-density (left) and high-density (right) HS films displayed in (*a*) (with colour code as indicated). The curves largely superpose for all four CXCL12*α* constructs, indicating that, for a given combination of HS and protein surface densities, the mechanical properties of the HS films are comparable. Representative data for HS film formation (grey) is given for comparison. (*c,d*) Mobile fractions and their diffusion constants of b-HS (bound to fl-SAv on SLBs) before incubation with CXCL12*α* constructs, after incubation with the proteins at equilibrium (native CXCL12*α*, CXCL12*α*(5–67) and locked dimer at 0.64 µM monomer equivalents, partial monomer at 3.8 µM), and after elution of respective protein from the solution phase, as indicated. The fluorescent label of fl-SAv was confirmed by QCM-D not to induce any measurable non-specific binding of any of the CXCL12*α* constructs (not shown). The mobility data confirm that all CXCL12*α* constructs can cross-link HS.

We tested the effect of binding of these constructs to low- and high-density HS films by QCM-D, and HS mobility in low-density HS films by FRAP. As with the wild-type, both mutants bound to HS films (figure 4*a*, blue curves), but not to the supporting SAv-monolayer (figure 4*a*, grey curves with triangle symbols). Binding to HS was distinct, however, with regard to the magnitude of the frequency shift at equilibrium and reversibility upon elution in buffer. The locked dimer exhibited enhanced and more stable binding, whereas binding was reduced and less stable for the partial monomer, as compared with native CXCL12*α*. These systematic variations reflect the importance of CXCL12*α* dimerization in stabilizing the interaction between the protein and HS [21].

Interestingly, both mutants also generated pronounced decreases in dissipation (figure 4*a*, red curves) upon binding to HS, albeit with different magnitudes. Parametric plots of the Δ*D/*−Δ*f* ratio as a function of −Δ*f* are shown in figure 4*b*. At a given HS density, the curves were very similar for all three protein constructs, except at the highest magnitudes of Δ*f*. For thin films, the Δ*D/*−Δ*f* ratio is proportional to the elastic compliance *J*′ [31] and thus a simple relative measure for softness, whereas −Δ*f* is a relative measure for the protein surface density. The plots illustrate that the softness of HS films reduces only marginally as the HS grafting density increases during HS film formation (i.e. from −Δ*f* = 0 to 28.5 ± 1 Hz), and that subsequent protein binding (cf. larger values of −Δ*f*) reduces the softness drastically and in a coverage-dependent manner. The fact that the Δ*D/*−Δ*f* versus −Δ*f* curves for protein binding are superimposed indicates that the mechanical properties (and hence the morphologies) of the HS films are comparable for a given HS surface density and protein concentration in the film, irrespective of the quaternary state of the employed protein. This implies that the differences in the magnitude of Δ*f* and Δ*D* at equilibrium are entirely because of differences in the affinity (i.e. the adsorbed amounts), but that the intrinsic propensity of CXCL12*α* to cross-link HS does not depend on protein oligomerization.

Complementary FRAP assays revealed that the partial monomer and locked dimer can effectively reduce the mobile fraction (figure 4*c*) and its diffusion constant (figure 4*d*), confirming that all CXCL12*α* constructs can indeed cross-link HS. However, an appreciable reduction in mobility for the partial monomer could only be observed after increasing the protein solution concentration (by sixfold). Moreover, after elution of residual partial monomer from the bulk solution with working buffer, the mobile fraction and its diffusion constant returned close to the level of a virgin HS film, whereas both parameters remained unaffected for the locked dimer. This demonstrates that an efficient cross-linking of the HS film requires a minimal protein concentration. Taken together, we conclude that the HS-induced CXCL12*α* dimerization [21,22] enhances protein binding, but that this dimeric structure is dispensable for HS cross-linking if the reduced affinity is compensated by an increased protein solution concentration.

CXCL12*α* mutants lacking the N-terminal lysine residue have been reported to display reduced affinity for HS based on surface plasmon resonance data [15,33], while nuclear magnetic resonance (NMR) analysis found no direct evidence of interaction with heparin-derived oligosaccharides [19,33]. We hypothesized that this amino acid, which forms the end of a rather flexible protein domain and is rather distant from all other amino acids known to be involved in HS binding [17], may be important for cross-linking. To test this, we studied an additional construct with a truncated amino acid sequence, i.e. a mutant that lacked the four N-terminal amino acids (CXCL12*α*(5–67); figure 1*a*). The magnitudes of the frequency shifts for this construct were comparable with native CXCL12*α* on high-density HS films and slightly reduced on low-density HS films (figure 4*a*), consistent with a rather weak contribution of the N-terminal lysine to protein binding. Importantly, the mutant also showed a negative dissipation shift, and the Δ*D/*−Δ*f* versus−Δ*f* curves for CXCL12*α*(5–67) and native CXCL12*α* at a given HS surface density (figure 4*b*) were indistinguishable. Moreover, FRAP results (figure 4*c,d*) confirmed that the mutation does not affect HS mobility. Taken together, these data indicate that the N-terminus is also dispensable for cross-linking, which is presumably consistent with its modest and/or transient interaction with HS [15,17,33].

### 3.5. Effect of CXCL12*α* on HS oligomers

**Figure 5.**
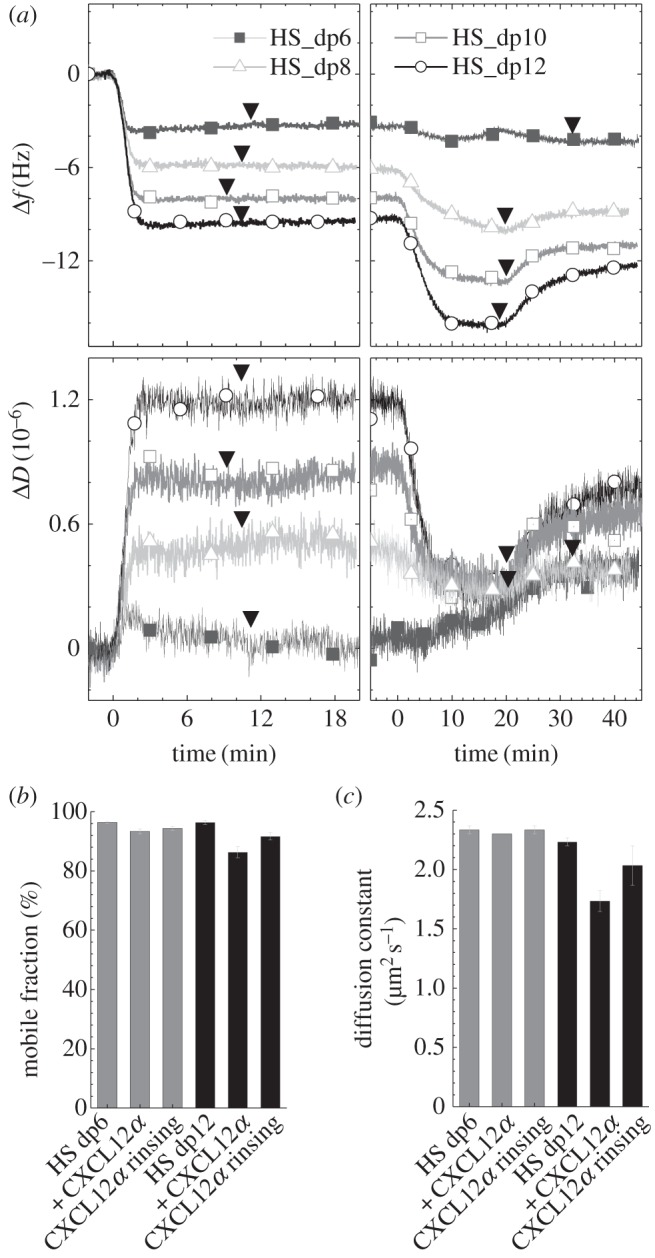
CXCL12*α* binding to and rigidification of films of oligomeric HS. (*a*) CXCL12*α* binding to monolayers of b-HS oligosaccharides of different lengths (as indicated; dp = degree of polymerization), immobilized on a SAv-monolayer on OEG (figure 2) was monitored by QCM-D to test the minimal length needed for the chemokine to bind and to cross-link HS. Injection of b-HS oligosaccharides (at 50 µg ml^−1^; left panels) and CXCL12*α* (at 0.64 µM; right panels) started at 0 min, and arrowheads indicate the start of rinsing with working buffer. Clear binding of CXCL12*α* is only observed for HS of dp8 (Δ*f* =−4 Hz) and larger, indicating that a hexasaccharide is not sufficiently long for protein binding. Pronounced dissipation decreases for HS as small as dp8 indicate that even films of oligomeric HS are rigidified. (*b,c*) Mobile fractions and their diffusion constants of b-HS oligosaccharides (bound to fl-SAv on SLBs) either bare or in the presence of 0.64 µM CXCL12*α*, as indicated. The moderate reduction in dp12 mobility suggests that oligosacharides can be cross-linked into relatively small clusters by CXCL12*α*.

Having established that CXCL12*α* cross-links HS, we next tested if there is a minimal length of HS chains required for cross-linking. CXCL12*α* binding to HS oligosaccharides of different size was analysed by QCM-D to determine the minimum number of saccharides necessary for CXCL12*α* binding and cross-linking (figure 5*a*). No response was observed on hexasaccharides (dp6), while clear binding was present on dp8, dp10 and dp12, confirming that an octasaccharide but not a hexasaccharide is sufficient for efficient binding, in agreement with the literature [15]. The dissipation decreased only slightly yet significantly (−0.1 × 10^−6^) for dp8, while films of dp10 and dp12 showed pronounced dissipation decreases upon CXCL12*α* binding. Clearly, the chemokine induced a rigidification of the oligosaccharide HS layers, suggesting that even rather short HS chains can be cross-linked.

Consistent with this interpretation, FRAP measurements on dp12 revealed significant decreases in the mobile fraction and its diffusion constant with CXCL12*α* (figure 5*b,c*). No significant effect was observed with dp6, as expected, demonstrating specificity of the assay. The effect of CXCL12*α* on the mobility of dp12 was, however, rather weak. This indicates that the oligosaccharides assemble into relatively small clusters with largely retained lateral mobility. In other words, longer HS chains are required for a sufficient amount of CXCL12*α* to bind to each chain and thus to induce effective cross-linking of many HS chains.

### 3.6. Effect of other HS-binding proteins on HS films

**Figure 6.**
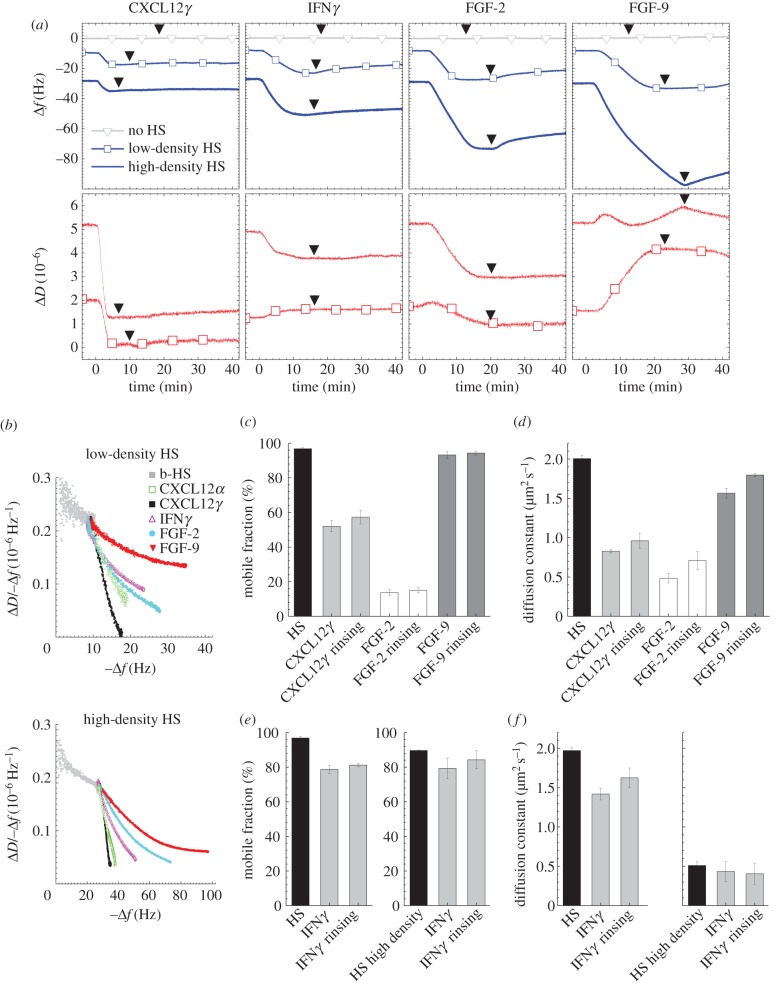
Correlation between structure and HS cross-linking propensity of HS-binding proteins. (*a*) QCM-D data for binding of proteins to HS films are displayed analogous to figure 4*a*. CXCL12*γ*, as CXCL12*α*, induced strong negative shifts in dissipation irrespective of HS film density; FGF-2, but not FGF-9, induced negative dissipation shifts irrespective of HS film density; for IFN*γ*, the dissipation decreased only on high-density HS films, indicating distinct, protein-specific degrees of HS film rigidification. (*b*) Parametric plot of Δ*D*/−Δ*f* for the protein-loaded HS film versus –Δ*f* for protein binding for the binding data on low-density (top) and high-density (bottom) HS films displayed in (*a*); the curves show that HS film rigidification depends on HS surface density, and protein type and coverage. (*c,d*) Mobile fractions and their diffusion constants of b-HS (bound to fl-SAv on SLBs at low surface density) either bare or in the presence of CXCL12*γ*, FGF-2 or FGF-9, as indicated. (*e,f*) Mobile fractions and their diffusion constants of b-HS (bound to fl-SAv on SLBs at low surface density (left graphs) and high surface density (right graphs)) either bare or in the presence of IFN*γ*, as indicated. The fluorescent label of fl-SAv was confirmed by QCM-D not to induce any measurable non-specific binding of any of the HS-binding proteins (not shown). Protein concentrations used throughout were 0.43 µM for CXCL12*γ*, 0.29 µM for IFN*γ*, 0.28 µM for FGF-2 and 0.17 µM for FGF-9. The mobility and rigidification data correlate, confirming that FGF-2 is a potent cross-linker whereas FGF-9 does not cross-link, that CXCL12*γ* cross-links HS film similarly to CXCL12*α* locked dimer, and that IFN*γ* is a rather poor cross-linker.

To test whether HS-cross-linking is unique to CXCL12*α*, we extended our study and systematically investigated the effect of several other HS-binding proteins, namely CXCL12*γ*, IFN*γ*, FGF-2 and FGF-9, on high- and low-density HS surfaces by QCM-D, and on low-density HS surfaces by FRAP (figure 6). The structures of all tested proteins are known and HS binding sites have been identified [23,34,36,37,43] (figure 1*b–e*). As expected, none of the proteins exhibited any significant non-specific binding to the SAv-monolayer (figure 6*a*, grey curves with triangle symbols).

Compared with CXCL12*α* (figure 1*a*), CXCL12*γ* (figure 1*b*) features 30 additional amino acids at the C-terminus, which are known to have HS-binding activity and enhance the affinity of CXCL12 for HS: dissociation constants *K*_D_ of 200 nM and 1.5 nM have been reported for the *α* and *γ* isoforms, respectively [23]. Indeed, CXCL12*γ* bound more stably and more rapidly than CXCL12*α* (figures 6*a* and 4*a*, respectively, blue curves). The decrease in dissipation for CXCL12*γ* was pronounced at low and high HS coverage (figure 6*a*, red curves). The Δ*D/*−Δ*f* versus −Δ*f* plots (figure 6*b*) confirm that this protein also has a strong propensity to rigidify the HS film. In these plots, differences between CXCL12*γ* and CXCL12*α* were small, albeit significant compared with the variations between CXCL12*α* and its mutants (figure 4*b*), suggesting that there are subtle differences in the morphology of the protein-loaded HS films. Nevertheless, CXCL12*γ* reduced the HS mobile fraction and its diffusion constant (figure 6*c,d*) somewhat more strongly than native CXCL12*α*, i.e. to a similar extent as the locked dimer of CXCL12*α* (figure 4*c,d*). We conclude that CXCL12*γ* is also a potent HS cross-linker and that this potency is enhanced by the HS binding stability.

IFN*γ* is a homodimeric cytokine known to strongly interact with HS (*K*_D_ ∼ 1 nM [39]). The known HS binding site is located at the C terminus and the two C termini in the homodimer are spatially separated (figure 1*c*). At present, it is not clear, if the two binding loci bind to a single or to two distinct HS chains. IFN*γ* readily bound to the HS films and binding was very stable as shown by the QCM-D frequency response (figure 6*a*). In high-density HS films, IFN*γ* induced a negative shift in dissipation (figure 6*a*) albeit with a reduced magnitude compared with CXCL12*α* (figure 4*a*). However, IFN*γ* generated a slight increase in dissipation in low-density HS films. FRAP (figure 6*e,f*, left plots) revealed that IFN*γ* induces only moderate reductions in the mobile fraction of HS (by 15%) and in the diffusion coefficient of this mobile fraction (by 25%). The lack of dissipation decrease and the weak reduction in HS mobility thus correlate, and indicate that IFN*γ* does not cross-link HS strongly, at least at low surface density. Under these conditions, the two HS binding sites on the IFN*γ* homodimer apparently bind within a single HS chain (intra-HS-chain bond).

To test if the decrease in dissipation at high HS surface density (figure 4*a*) is an indicator for the formation of inter-HS-chain bonds by IFN*γ* when HS chains are densely packed, we performed additional FRAP measurements at high HS surface densities (figure 6*e,f*, right plots). To this end, the fraction of biotinylated lipids used to form the SLB was increased (from 0.5 to 5%) to enable formation of a dense fl-SAv-monolayer. Under these conditions, the lateral mobility of the bare HS films was largely retained (i.e. the mobile fraction was only slightly reduced, to 90%) although crowding of fl-SAv entailed a marked reduction of the diffusion constant (from 2 µm^2^ s^−1^ to 0.5 µm^2^ s^−1^). Interestingly, the mobile fraction as well as its diffusion constant decreased only weakly in the presence of IFN*γ* (by 12% and 20%, respectively). This indicates that the IFN*γ* homodimer prefers to form intra-HS rather than inter-HS-chain bonds even at high HS concentrations, and supports the previously proposed model in which IFN*γ* binds to two adjacent N-sulfated domains along a single HS chain [44].

FGF-2 and FGF-9 were selected because of their well-characterized HS binding sites (figure 1*d,e*). FGF-2 has three HS binding sites, of which two are located on the same face and the third on the opposite face of the protein [37,43,45,46]. By contrast, only one (rather extended) HS binding site has been identified for FGF-9 [25]. FGF-2 and FGF-9 were reported to have affinities of 10 and 620 nM, respectively, to heparin dp8 (i.e. a representative of high-affinity binding sites on HS) [25,47]. Both FGFs bound readily to HS films (figure 6*a*), as expected. The frequency shifts on high-density HS films exceeded those observed for the previously investigated chemokines (figures 4 and 6), indicating extensive binding. FGF-2 generated pronounced decreases in dissipation for high-density and low-density HS films. By stark contrast, the dissipation remained largely unchanged and increased drastically, respectively, for FGF-9. This contrast is also apparent in the Δ*D/*−Δ*f* versus −Δ*f* plots (figure 6*b*), where the curves for FGF-9 are located above the curves for FGF-2 irrespective of the HS surface density, thus indicating that FGF-2 is more potent in rigidifying HS films. FRAP revealed a drastic reduction (by 80%) in the mobile fraction with FGF-2 (figure 6*c*), i.e. this protein essentially immobilized HS. FGF-9, on the other hand, did not affect the mobile fraction at all (figure 6*c*) and the diffusion constant of the mobile fraction was only weakly affected (figure 6*d*).

Clearly, FGF-2, but not FGF-9, has a strong propensity to cross-link and to rigidify HS films. In the light of the distinct structural features of these two growth factors, we propose that FGF-2 cross-links HS by accommodating at least two different chains simultaneously in its multiple HS binding sites, whereas only one HS chain at a time can bind to the extended binding site on FGF-9. The results with FGFs highlight that not all HS-binding proteins cross-link HS and that the cross-linking propensity can vary distinctly among proteins of the same family.

## 4. Discussion

### 4.1. What are the molecular mechanisms behind HS cross-linking?

One may argue that a protein with an HS-binding surface large enough to accommodate more than one HS chain should be able to cross-link HS. Yet, we found the extension of the HS-binding surface alone to be a poor predictor of a protein's cross-linking propensity. This is illustrated by the limited cross-linking propensity of the IFN*γ* homodimer (figure 6), and also by the negligible effects of the elongated C-terminal of CXCL12*γ*, compared with CXCL12*α*, on HS film rigidification and cross-linking (figures 4 and 6). Apparently, the formation of multiple bonds with the same HS chain is more favourable in these cases than the inter-connection of several distinct HS chains.

FGF-2, by contrast, exhibited strong cross-linking activity (figure 6). A detailed inspection of the protein's surface reveals that the three HS-binding patches containing basic amino acids are separated from each other by acidic and hydrophobic amino acids. Such HS-repelling rims are not present in any of the other proteins tested. From the correlation with our experimental data, we thus propose multiple HS-binding patches separated by HS-repelling borders as a distinct structural feature conducive to HS cross-linking.

Mutation of the primary binding site reduces binding of FGF-2 to HS substantially [48], i.e. the affinities of the secondary HS binding sites on FGF-2 are rather weak. Yet, FGF-2 apparently is a potent HS cross-linker. This effect is not surprising if one takes into consideration that, once FGF is sequestered into the matrix through its primary high-affinity binding site, the local concentration in HS is high such that even weak interactions can occur frequently. Thus, the example of FGF-2 illustrates how rather weak secondary binding sites can fulfil functions.

CXCL12 is also a potent HS cross-linker (figures 4–6), yet the molecular mechanism of cross-linking must be different since this protein does not feature several clearly separated binding sites. It is instructive to consider the quaternary structure of this protein. Upon HS binding, CXCL12 readily forms homodimers through the association of β-sheets [15], but our tests with partial monomer and locked dimer (figure 4) demonstrated that this ‘β-sheet’ dimer is not directly involved in HS cross-linking. Crystallographic studies [49] though revealed that CXCL12*α* can form another homodimer through the association of two N-termini, analogous to what is commonly observed for chemokines of the CC family [50], although the functional significance of the ‘N-terminal’ dimer has so far remained unclear.

We propose that β-sheet and N-terminal dimers coexist in the HS matrix, potentially forming dimers of dimers. In this scenario, the two dimerization mechanisms would have distinct functions, i.e. dimerization through β-sheets enhances the affinity of the protein for HS whereas dimerization through N-termini induces HS cross-linking. Our experimental data are fully consistent with such a scenario. In particular, arginines at positions 8 and 12 were found to be involved in the formation of the N-terminal dimer [49]. These are present in all mutants (including the truncated CXCL12*α*(5–67) form), and it is thus not surprising that all our CXCL12*α* constructs exhibited a similar propensity to rigidify and cross-link HS films once the differences in affinity were adjusted for (figure 4*b–d*). Moreover, an N-terminal dimer can also readily cross-link short HS oligosaccharides (figure 5), whereas such an effect would be difficult to explain with β-sheet dimers alone: in the current binding model, dp8 is just long enough to fit the HS-binding interface in the β-sheet dimer [19]; it would be conceivable that a single dp8 binds two β-sheet dimers (i.e. one on each face of the oligosaccharide), but not the opposite. Future studies with other CXCL12*α* mutants should be useful to test if the arginines at positions 8 and 12 are indeed crucial for dimerization-mediated cross-linking and how HS binding [19,33] and CXCL12*α* oligomerization interplay to promote cross-linking. In this regard, it is notable that many chemokines form oligomers, when free in solution or upon binding to GAGs [51]. It will thus also be interesting to investigate how the oligomerization of other chemokines correlates with their propensity for cross-linking GAGs.

### 4.2. The methodological approach presented in this study is novel

HS films as model matrices present HS at controlled orientation and lateral mobility and at tuneable surface density, thus enabling supramolecular interaction studies under well-defined conditions. The two characterization techniques, QCM-D and FRAP, provide complementary information and together enable identification of the protein's binding and cross-linking activity. Specifically, QCM-D provides information about binding kinetics, and about HS/protein film morphology (thickness) and rigidity, whereas FRAP enables quantification of the lateral mobility of HS chains. The assay does not require any labelling of the protein and is thus broadly applicable to assess the propensity of proteins to cross-link HS and other GAGs. In particular, some extracellular signalling proteins are known to bind several types of GAGs (e.g. CXCL12*γ* [23,38] or IFN*γ* [38–40]) and it will thus be interesting to probe if the propensity of a given protein to cross-link is specific to a particular GAG type.

GAG-on-chip devices are increasingly used to probe the interaction of GAGs with proteins. On such devices, the extent of protein-mediated GAG cross-linking will depend sensitively on the presentation and surface density of GAGs. As a consequence, the binding behaviour of proteins may also vary strongly, calling for care in the interpretation of the read out and comparison of data between different GAG-on-chip-based assays. The method developed here should be very useful to evaluate how GAG presentation and surface density affect binding.

### 4.3. What is the functional relevance of HS cross-linking by extracellular signalling proteins?

Cross-linking of HS requires the spatial proximity of HS chains. This criterion was met on average in our well-defined model matrices within the range of HS surface densities employed. Based on the typical length of HS chains and the typical density of HS-bearing proteoglycans (PGs), Yanagishita & Hascall [14] estimated that the ensemble of HS chains on cells can readily explore the entire cell surface. This implies that neighbouring HS chains can meet, and HS cross-linking thus may also be a frequent phenomenon at the cell surface and in extracellular matrix. The distribution of HS, however, may not be homogeneous across the cell surface [52] and may vary across cell types and states. This implies that the local HS density can vary over a large range, and that cross-linking may be confined to specific locations. It is thus possible that HS cross-linking is spatio-temporally controlled through the expression of HS and the sequestration of chemokines or growth factors in the course of specific biological processes (e.g. angiogenesis [53], inflammation [54], cell proliferation [6,47,55]). This may have consequences at different levels.

On the level of the matrix, the proteins can promote changes in structure that parallel their signalling activity. The ensuing changes in physical properties of peri- and extracellular matrices, such as permeability, rigidity or thickness, may elicit a range of additional cellular responses. For example, a reduction in the thickness of pericellular coats may facilitate intercellular contacts through membrane-bound cell adhesion receptors/ligands [56], or the cross-linking of HS displayed by two distinct pericellular coats could be important in the initial stage of cell–cell adhesion. Moreover, changes in the rigidity of the cellular glycocalyx through HS cross-linking may provide a physical cue that guides the behaviour of cells.

On the local scale, cross-linking of HS could promote clustering of cell-surface PGs to which the HS chains are attached, thereby activating signalling. Clustering of the HSPG syndecan-4, for example, is important for the binding to and activation of protein kinases which ultimately determine the assembly of focal adhesions and the organization of the actin cytoskeleton [57]. In this regard, it has been demonstrated that a syndecan-4 dimer requires a minimum of four HS chains to be functional, whereas a mutated form of syndecan-4 with a single HS chain was not functional unless a cluster of multiple syndecan-4 dimers was formed. This suggests that multiple HS chains must associate in the presence of a ligand, to form a signalling unit [58]. In this scenario, HS-cross-linking proteins would elicit signalling activity in a way that has thus far not been appreciated. Moreover, the interaction between FGFs and syndecans has been demonstrated to promote their clustering, activation of protein kinase C*α*, translocation to cholesterol-rich membrane domains and eventually internalization and transfer of FGF-2 to the nucleus [59–62]. Thus, ligands that do not cause cross-linking of HS chains, such as IFN*γ* and FGF-9, may not be able to activate these parallel, HS-specific signalling pathways. A further HS-dependent pathway that may be activated by ligands that cross-link HS chains on syndecans is the formation of exosomes [63]. Future studies comparing the effect of proteins that cross-link HS (e.g. FGF-2, CXCL12*α* or CXCL12*γ*) with those that do not (e.g. IFN*γ* or FGF-9) would provide a direct test if HS cross-linking is important for exosome formation and other HS-specific signalling pathways. Last but not least, the proteins themselves would also be affected by HS cross-linking, in that the attachment through multiple binding sites reduces their mobility. This may contribute, for example, to the substantial fraction of FGF-2 that is observed to undergo confined, rather than diffusive motion in pericellular matrix [52].

## 5. Conclusion

In summary, we have demonstrated that extracellular signalling proteins can cross-link GAGs and propose that several binding sites, well separated either through GAG-repellent borders on the protein's surface (e.g. FGF-2) or through spatial separation in quaternary protein structures (e.g. N-terminal CXCL12 dimers), are required for GAG cross-linking. This prediction can now readily be tested with other GAG-binding proteins using the here-presented GAG cross-linking assay. The ability of extracellular signalling proteins to influence matrix organization and physico-chemical properties implies that the functions of these proteins may not simply be confined to the activation of cognate cellular receptors. This may have far-reaching implications for cell–cell and cell–matrix communication, and our predictions can be tested in future cell and *in vivo* assays.
